# Genetic and phenotypic characterization of Parkinson’s disease at the clinic-wide level

**DOI:** 10.1038/s41531-024-00690-6

**Published:** 2024-05-03

**Authors:** Thomas F. Tropea, Whitney Hartstone, Noor Amari, Dylan Baum, Jacqueline Rick, Eunran Suh, Hanwen Zhang, Rachel A. Paul, Noah Han, Rebecca Zack, Eliza M. Brody, Isabela Albuja, Justin James, Meredith Spindler, Andres Deik, Whitley W. Aamodt, Nabila Dahodwala, Ali Hamedani, Aaron Lasker, Howard Hurtig, Matthew Stern, Daniel Weintraub, Pavan Vaswani, Allison W. Willis, Andrew Siderowf, Sharon X. Xie, Vivianna Van Deerlin, Alice S. Chen-Plotkin

**Affiliations:** 1 Department of Neurology, Perelman School of Medicine at the University of Pennsylvania, Philadelphia, PA USA; 2 Department of Pathology and Laboratory Medicine, Philadelphia, PA USA; 3 Department of Ophthalmology, Perelman School of Medicine at the University of Pennsylvania, Philadelphia, PA USA; 4 Parkinson’s Disease Research, Education and Clinical Centers (PADRECC), Philadelphia Veterans Affairs Medical Center, Philadelphia, PA USA; 5 Department of Psychiatry, Perelman School of Medicine at the University of Pennsylvania, Philadelphia, PA USA; 6 Department of Biostatistics, Epidemiology, and Informatics, Perelman School of Medicine at the University of Pennsylvania, Philadelphia, PA USA; 7 Present Address: Abramson Cancer Center, Perelman School of Medicine at the University of Pennsylvania, Philadelphia, PA USA

**Keywords:** Parkinson's disease, Genetic testing, Translational research, Neurological manifestations

## Abstract

Observational studies in Parkinson’s disease (PD) deeply characterize relatively small numbers of participants. The Molecular Integration in Neurological Diagnosis Initiative seeks to characterize molecular and clinical features of every PD patient at the University of Pennsylvania (UPenn). The objectives of this study are to determine the feasibility of genetic characterization in PD and assess clinical features by sex and *GBA1/LRRK2* status on a clinic-wide scale. All PD patients with clinical visits at the UPenn PD Center between 9/2018 and 12/2022 were eligible. Blood or saliva were collected, and a clinical questionnaire administered. Genotyping at 14 *GBA1* and 8 *LRRK2* variants was performed. PD symptoms were compared by sex and gene groups. 2063 patients were approached and 1,689 (82%) were enrolled, with 374 (18%) declining to participate. 608 (36%) females were enrolled, 159 (9%) carried a *GBA1* variant, and 44 (3%) carried a *LRRK2* variant. Compared with males, females across gene groups more frequently reported dystonia (53% vs 46%, *p* = 0.01) and anxiety (64% vs 55%, *p* < 0.01), but less frequently reported cognitive impairment (10% vs 49%, *p* < 0.01) and vivid dreaming (53% vs 60%, *p* = 0.01). *GBA1* variant carriers more frequently reported anxiety (67% vs 57%, *p* = 0.04) and depression (62% vs 46%, *p* < 0.01) than non-carriers; *LRRK2* variant carriers did not differ from non-carriers. We report feasibility for near-clinic-wide enrollment and characterization of individuals with PD during clinical visits at a high-volume academic center. Clinical symptoms differ by sex and *GBA1*, but not *LRRK2*, status.

## Introduction

Parkinson’s disease (PD) affects >6 million people worldwide, making it the second most common neurodegenerative disease, affecting 1–3% of people over age 65^1–3^. The disorder is characterized by bradykinesia plus tremor or rigidity^4^; PD can also affect mood, cognition, sleep, and autonomic function, collectively referred to as non-motor symptoms^5^.

The occurrence of motor complications such as fluctuations in medication responsiveness, dyskinesia, and dystonia, as well as non-motor symptoms, varies from person to person and over the disease course. The frequency and impact of motor and non-motor symptoms of PD have been demonstrated in research cohorts, where they have been shown to vary with sex, age, and with variants in *GBA1* or *LRRK2*, two common PD risk genes^6–13^.

The real-world experience of patients with PD may differ from what has been described in research studies, which may suffer from selection bias, recruiting those individuals with the fewest barriers to participation^14–16^. Indeed, minorities and women are under-represented in PD research cohorts, consistent with this bias^17^. A clinic-wide approach to assessments of motor and non-motor features, capturing all patients at the time of their clinical visit, may address this selection bias to give a more accurate real-world accounting of PD symptom frequency. We hypothesized that interest in research enrollment would be high among PD patients when typical barriers to enrollment such as transportation and time were reduced, and that sex- and gene-defined groups would report different frequencies in motor and non-motor symptoms of PD and medication use at the clinic-wide level.

Here we report the results of a clinic-wide research recruitment at a single academic center. The Molecular Integration in Neurological Diagnosis (MIND) initiative aimed to approach every patient with PD seen at the University of Pennsylvania (UPenn) Parkinson’s Disease and Movement Disorders Center (PDMDC). Patients were approached for enrollment at their clinical visit, and enrolled participants provided information about their PD symptoms, a blood or saliva sample, as well as optional consent to allow for access to their medical record, recontact in the future for additional studies for which they may be eligible, and future use of their blood or saliva samples for additional research. We present our findings from 1689 participants enrolled, representing 82% of the 2063 patients approached for enrollment. Variants in *GBA1* and *LRRK2* were identified, and clinical symptoms were analyzed by sex and gene status. Our study demonstrates feasibility of near-clinic-wide research enrollment and clinical and genetic characterization.

## Results

### Participant enrollment

**Fig. 1:**
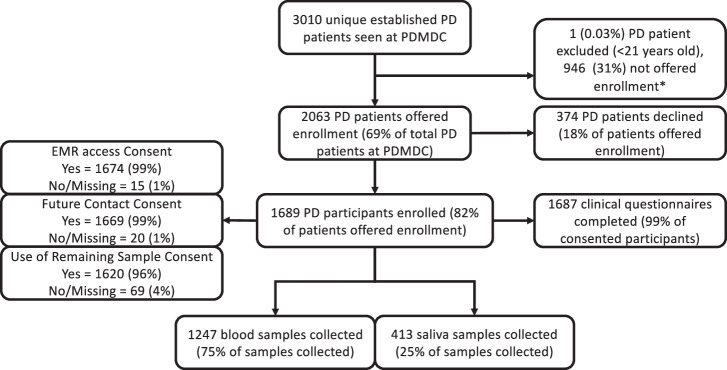
Summary of enrollment in the molecular integration in neurological diagnosis (MIND) Parkinson’s Disease cohort. PDMDC = Parkinson’s Disease and Movement Disorders Center at the University of Pennsylvania. EMR = Electronic Medical Record. *Participants were not offered enrollment due to logistical reasons (staffing, room availability, unsuccessful patient contact within window followed by patient death or no longer following at UPenn, visits too late in the day for same-day sample processing, among others).

Participants were enrolled between September 7, 2018, and December 23, 2022. During this period, 19 Movement Disorders neurologists or nurse practitioners completed in-person or telemedicine patient visits for 3010 unique PD patients at the PDMDC. Of 3010 established PD patients, 1 (0.03%) did not meet enrollment criteria (<21 years old), and 946 (31%) were not approached due to logistical reasons (staffing, room availability, unsuccessful patient contact within window followed by patient death or no longer following at UPenn, visits too late in the day for same-day sample processing, among others). 2063 patients were successfully captured at the time of their visit and offered enrollment in the MIND cohort, representing 69% of the UPenn PDMDC PD population. Three hundred and seventy-four patients (18% of patients offered enrollment) declined enrollment, while 1689 patients (82% of PD patients offered enrollment or 56% of all established PD patients) consented to enrollment. A summary of enrollment as well as clinical data, blood and saliva collection, and optional consent can be seen in Fig. 1.

### Demographics and cohort characteristics

Table 1 provides a summary of cohort demographics stratified by gene status. Among 1689 PD participants, 159 *GBA1* and 44 *LRRK2*-variant carriers were identified. Three individuals (<1% of cohort) carried variants in both *GBA1* and *LRRK2* (2 *GBA1* N409S/*LRRK2* G2019S carriers, and one *GBA1* N409S/*LRRK2* homozygous G2019S variant carrier). Three (<1%) *LRRK2* G2019S homozygotes were identified (including the *GBA1* N409S co-carrier*/LRRK2* G2019S), and 3 (<1%) *GBA1* homozygotes were identified (1 E365K/E365K, 1 N409S/N409S, 1 T408M/T408M). One participant carried the *GBA1* H294Q and D448H variants, and 1 Rec1 carrier (carrying the L483P, A495P, and Val499= variants) was identified. See Supplementary Fig. 1 and Supplementary Table 2 for summaries of *GBA1* and *LRRK2* testing results.

**Table 1:** Cohort demographics

	Whole Cohort	*GBA1*/*LRRK2* Negative^b^	*GBA1* Carriers	*p*	*LRRK2* Carriers	*p*
*N* (%)	1689	1483	159^c^		44^c^	
Variant, *n* (%)						
* GBA1* Risk			94 (59)			
* GBA1* Mild			42 (26)			
* GBA1* Severe			23 (14)			
* LRRK2* G2019S					41 (93)^d^	
Sex, *n* (%)^a^						
Female	608 (36)	517 (35)	67 (42)	0.081	**23 (51)**	**0.048**
Male	1080 (64)	965 (65)	92 (58)		**21 (49)**	
Ethnicity, *n* (%)						
Hispanic or Latino	34 (2)	31 (2)	2 (1)	nt	1 (2)	nt
Not Hispanic or Latino	1654 (98)	1451 (98)	157 (99)		43 (98)	
Unknown or not reported	1 (<1)	1 (<1)	0 (0)		0 (0)	
Race, *n* (%)						
White	1532 (91)	1334 (90)	155 (98)	nt	40 (91)	nt
Black or African American	78 (5)	76 (5)	2 (1)		0 (0)	
Am Indian/Alaska Native	1 (<1)	1 (<1)	0 (0)		0 (0)	
Asian	56 (3)	54 (4)	0 (0)		2 (4)	
More than one race	9 (<1)	6 (<1)	2 (1)		1 (2)	
Other	5 (<1)	4 (<1)	0 (0)		1 (2)	
Unknown or not reported	8 (<1)	8 (1)	0 (0)		0 (0)	
Age, mean (SD)						
At diagnosis	62 (11)	62 (11)	61 (10)	0.215	61 (11)	0.347
At enrollment	69 (9)	69 (9)	**67 (9)**	**0.049**	70 (9)	0.683
Clinical Presentation at Onset						
Tremor	865 (51)	764 (52)	77 (49)	0.482	22 (50)	0.482
Gait Disorder	191 (11)	170 (11)	18 (11)		2 (5)	
Mixed	120 (7)	101 (7)	16 (10)		3 (7)	
Neither	513 (31)	448 (30)	48 (30)		17 (38)	
1st Degree Family History						
None	1401 (83)	1241 (84)	128 (80)	0.225	**30 (68)**	**0.002**
PD	155 (9)	122 (8)	20 (13)		**12 (27)**	
Other NDD	108 (6)	99 (7)	8 (5)		**1 (2)**	
Both	25 (2)	21 (1)	3 (2)		**1 (2)**	

*GBA1* (*N* = 3) and *LRRK2* (*N* = 2) homozygotes and 1 individual carrying two different variants in *GBA1* were included.

^a^1 missing/unreported. *Nt* not tested.

^b^Includes 28 cases without DNA available. Box indicates corrected *p* < 0.05 relative to GBA1/LRRK2 Negative group. *P* values after Benjamini-Hochberg multiple testing correction are reported.

^c^Three individuals carried variants in both *GBA1* and *LRRK2* (all were *GBA1* N409S/*LRRK2* G2019S) and are excluded. *NDD* neurodegenerative disease.

^d^3 LRRK2 G2385R carriers were identified (6% of LRRK2 carriers).

Significant differences among groups are indicated in bold type.

### GBA1 and LRRK2 Variant Carriers: Sex and Family History

While the entire MIND cohort was enriched with males (64% of whole cohort), LRRK2 G2019S carriers with PD differed in the proportion of males to females compared to non-carriers (exact *p* = 0.024, corrected *p* = 0.048); 49%, 95% CI 26–93, of *LRRK2* G2019S carriers were males. *LRRK2* G2019S carriers (*N* = 44) were more likely to report having a 1st degree family member with PD (exact *p* = 0.001, corrected *p* = 0.002) than *GBA1* non-carriers or *LRRK2* non-carriers (*N* = 1483). *GBA1* carriers had a lower age at study enrollment compared to *GBA1* non-carriers or *LRRK2* non-carriers (*z* = −2.42, exact *p* = 0.025, corrected *p* = 0.049). No differences were detected between *GBA1* carriers and *GBA1* non-carriers or *LRRK2* non-carriers in frequency of sex, age at diagnosis, presentation at onset, and family history of neurodegenerative diseases. No differences were detected between *LRRK2* carriers and *GBA1* non-carriers or *LRRK2* non-carriers in age at diagnosis or enrollment or clinical presentation at onset (Table 1).

### Parkinson’s disease motor and non-motor features on a clinic-wide scale

**Fig. 2:**
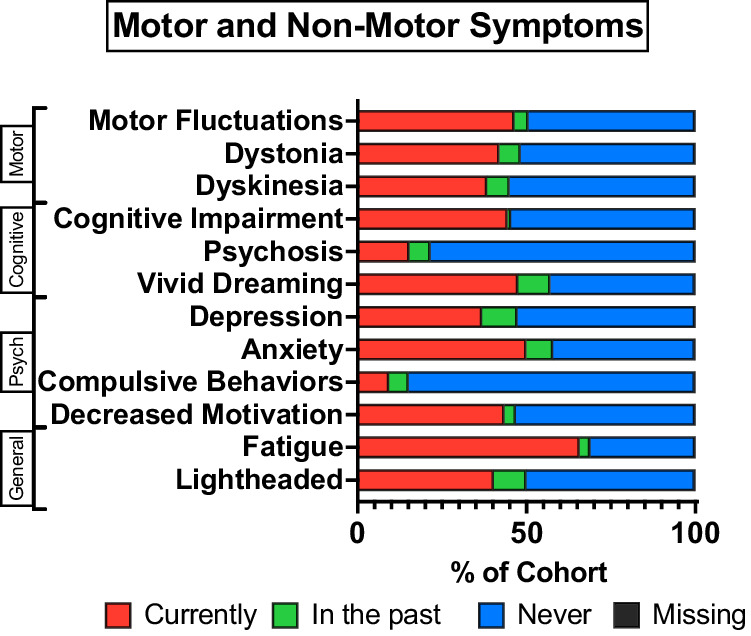
Motor and non-motor Parkinson’s disease symptoms. Self-reported motor and non-motor symptoms from *N* = 1689 Parkinson’s Disease participants. Two missing due to incomplete clinical questionnaire. Psych, Psychological.

Participants were asked to report on their current or prior history of 12 different motor and non-motor symptoms of PD. Among enrolled participants, 1680 (99% of cohort) responses were collected. The most frequently reported symptoms were fatigue (69% currently experiencing), anxiety (50%), and vivid dreaming (47%), while the least common symptoms were compulsive behaviors (9%), psychosis (15%) and depression (37%, Fig. 2).

### Females and males differ in the frequency of motor and non-motor symptoms

**Fig. 3:**
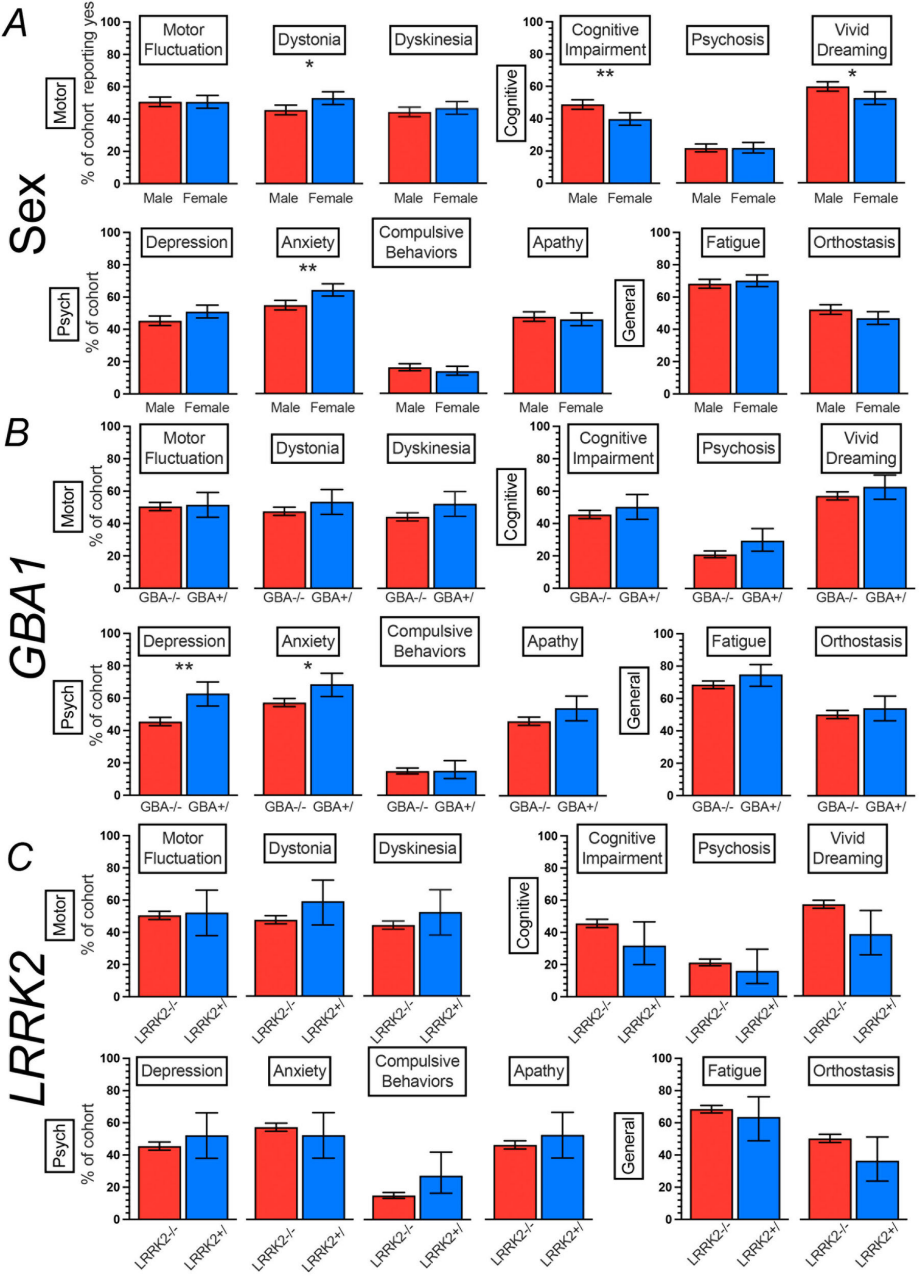
Motor and non-motor Parkinson’s disease symptoms by sex, *GBA1*, and *LRRK2* variant status. Responses grouped as ever (currently or in the past) or never experiencing a symptom. Heterozygote and homozygote carriers are combined. *GBA*/*LRRK2* co-carriers omitted (*N* = 3). *p < 0.05, **p < 0.01. Error bars represent 95% CI. Panel **A** compares sexes, Panel **B** compares groups by *GBA1* status, and Panel **C** compares groups by *LRRK2* status.

Females (*N* = 608), accounting for 36% of the MIND cohort, reported a higher frequency of current or prior dystonia (53%, 95% CI = 49–57, exact *p* = 0.003, corrected *p* = 0.012) and anxiety (64%, 95% CI = 60–68, exact *p* = 0.0002, corrected *p* = 0.002) compared to males (*N* = 1080, 46%, 95% CI = 43–49 and 55%, 95%CI = 52–58, respectively). Additionally, females reported a lower frequency of current or past cognitive impairment (40%, 95% CI = 36–44, exact *p* = 0.0003, corrected *p* = 0.002) and vivid dreaming (53%, 95% CI = 49–57, exact *p* = 0.004, corrected *p* = 0.012) compared to males (49%, 95% CI = 46–52, and 60%, 95% CI = 57–63, respectively). The frequencies of depression and lightheadedness did not differ between females and males after correction for multiple testing (exact *p* = 0.03 and 0.03, corrected *p* = 0.06 and 0.07, respectively). All other motor and non-motor symptom frequencies did not differ between females and males (Fig. 3A).

### GBA1 variant carriers report a higher frequency of depression and anxiety than non-carriers

Carriers of variants in *GBA1* (*N* = 159), accounting for 9% of the MIND cohort, reported a higher frequency of current or past depression (63%, 95% CI = 55–70, exact *p* = 0.00003, corrected *p* = 0.0004) or anxiety (69%, 95% CI = 61–75, exact *p* = 0.006, corrected *p* = 0.036), compared to *GBA1*/*LRRK2* non-carriers (*N* = 1483, 46%, 95% CI = 43–48, and 57%, 95% CI = 55–60, respectively). The frequency of psychosis did not differ between *GBA1* carriers and those not carrying a variant in *GBA1* after correcting for multiple testing (exact *p* = 0.02, corrected *p* = 0.054). Other motor and non-motor symptoms did not differ between *GBA1* carriers and those not carrying a variant in *GBA1* (Fig. 3B). Among *GBA1* variant carriers, no differences in motor and non-motor symptoms were detected comparing individuals carrying risk, mild, or severe *GBA1* variants (categories detailed in Supplementary Table 1 and Supplementary Fig. 2).

### LRRK2 variant carriers did not differ from non-carriers in motor and non-motor symptoms

No differences in motor and non-motor symptoms were detected between individuals carrying the G2019S *LRRK2* variant (*N* = 44, 3% of cohort) and those not carrying a variant in *GBA1* or *LRRK2* (*N* = 1483, Fig. 3C). The frequency of vivid dreaming comparing *LRRK2* variant carriers with *GBA1*/*LRRK2* non-carriers did not differ after correcting for multiple testing (exact *p* = 0.02, corrected *p* = 0.20).

### Use of common PD medications on a clinic-wide scale

All active prescriptions for PD-related medications at the enrollment visit were extracted from the medical record (*N* = 1674, 15 participants did not consent to future access to their medical record). Medications were grouped by mechanism of action, comprising all formulations of levodopa, dopamine agonists, monoamine oxidase-B inhibitors, catechol-o-methyltransferase inhibitors, anticholinergics, amantadine, adenosine A_2A_ antagonists, acetylcholinesterase inhibitors, and antipsychotics (including pimavanserin, quetiapine, and clozapine). Females were less likely to be prescribed a PD-related medication (8%, 95% CI = 6–11 of females not taking a PD medication, compared to 5%, 95% CI = 4–6 of males, exact *p* = 0.006, corrected *p* = 0.030), while males were prescribed acetylcholinesterase inhibitors more frequently than females (12%, 95% CI = 10–14 versus 5%, 95% CI = 4–7, respectively, exact *p* = 0.000006, corrected *p* = 0.00006). The prescription frequencies of all other medications did not differ between males and females, or between *GBA1* or *LRRK2* variant carriers and non-carriers (Table 2).

**Table 2:** Medication summary

	Whole Cohort	Male	Female	*GBA1*/*LRRK2* Negative	*GBA1* Carrier	*LRRK2* Carrier
*N* (%)	1674^a^	1070	604	1469	158^b^	44^b^
None	104 (6)	**53 (5)**	**51 (8)**	88 (5)	15 (1)	1 (<1)
Levodopa	1455 (87)	944 (88)	511 (85)	1281 (87)	134 (85)	38 (86)
DA agonist	356 (21)	219 (21)	137 (23)	310 (21)	28 (18)	17 (39)
MAOB-I	462 (28)	311 (29)	151 (25)	409 (28)	36 (23)	16 (36)
COMT Inh	86 (5)	58 (5)	28 (5)	73 (5)	9 (6)	4 (9)
Anticholinergic	31 (2)	23 (2)	8 (1)	26 (2)	5 (3)	0 (0)
Amantadine	327 (20)	201 (19)	126 (21)	288 (20)	27 (17)	11 (25)
A2A Ant	10 (1)	8 (1)	2 (<1)	9 (1)	0 (0)	1 (2)
AChEI	159 (10)	**127 (12)**	**32 (5)**	136 (9)	22 (14)	1 (2)
Antipsychotic	86 (5)	51 (5)	35 (6)	73 (5)	11 (7)	2 (5)

^a^N = 15 did not permit access to the electronic medical record.

^b^Three individuals carried variants in both *GBA1* and *LRRK2* (all were *GBA1* N409S/*LRRK2* G2019S) and are excluded. *DA* Dopamine, *MAOB-I* Monoamine oxidase B inhibitor, *COMT* catechol-o-methyltransferase, *A2A Ant* Adenosine A_2A_ receptor antagonist, *AChEI* Acetylcholinesterase inhibitor. Box indicates corrected *p* < 0.05.

Significant differences among groups are indicated in bold type.

## Discussion

Here we report the summary results of the MIND PD Cohort Study, which aimed to characterize molecular and clinical features of every PD patient in a large academic movement disorders center. A total of 2063 of 3010 unique PD patients who received care at the PDMDC between September 7, 2018, and December 23, 2022, were successfully approached at their clinical visit, and 1689 (82% of those approached) enrolled in the MIND cohort. The overwhelming majority of enrollees consented to access to the EMR (99% of 1689 enrolled), contact for future studies and possible genetic disclosure (99% of 1689 enrolled), and use of biosamples for future studies (96% of 1689 enrolled). Among enrollees, 159 (9%) harbored a *GBA1* variant, and 44 (3%) harbored a *LRRK2* variant.

Despite the unexpected transition of an active movement disorders practice to telemedicine-based visits during the COVID-19 pandemic, our all-comers approach continued to be highly efficient for enrolling participants, minimizing selection bias based on sex or self-described race. Continuing the trends reported in our interim analysis^18^, the percentage of participants who were female (36%) and those who identified as non-White (9%) were significantly higher than for prior genetic research studies from our same clinical center pre-dating this all-comers-enrollment strategy (32% female and <4% non-White in prior studies)^19^. Our findings suggest that a clinic-wide approach to participant capture at the time of the office visit minimizes barriers to research participation, mitigating selection bias. With 82% of all PD individuals approached consenting to enrollment in a single-visit, our study also provides evidence for widespread interest in research participation when barriers are reduced.

The existing landscape of PD cohort studies comprises “deep and narrow” cohorts vs. “shallow and wide” cohorts. In the former situation, exemplified by the international Parkinson’s Progression Marker Initiative (PPMI) De Novo PD cohort^20^, the Penn-based U19 cohort^21^, and others^22–24^, a small fraction of a total clinic population is extensively characterized. In the latter situation, exemplified by Fox Insight^25^ or the 23andMe PD cohort^26^, thousands of individuals are enrolled, but PD status is not clinically confirmed and information such as medication use is self-reported only. The MIND cohort is unique in capturing clinically confirmed PD patients, with EMR-confirmed medication use, while maintaining broad capture of a total clinical population. Indeed, of 3010 possible PD patients who received clinical care from 19 providers over a span of 52 months, the MIND study enrolled 1689 (56% of total clinic).

As a consequence, the MIND study provides a global picture of PD at the clinic-wide level, highlighting intriguing sex- and gene-based differences. For example, our finding that women with PD are more likely to report anxiety, but less likely to report cognitive impairment or vivid dreaming than men extends prior work suggesting sex-based differences in PD non-motor symptoms^7–9^, reviewed in^27,28^. Similarly, we find significant sex differences comparing PD individuals who do versus do not carry the *LRRK2* G2019S variant. Our finding supports prior work in targeted populations enriched for *LRRK2* variants^29–31^, suggesting sex-based differences in *LRRK2*-associated PD (or, more precisely, lack of sex-based differences generally observed in PD in this genetically-defined group). We note, however, that an important consideration in these cases is the breadth of our current study. Specifically, the sex-based differences in *LRRK2* G2019S PD are found in a clinic-wide study vs. one designed to evaluate a targeted genetic population. Similarly, prior reports of sex-based differences in symptoms among PD individuals have generally studied many fewer patients (ranging from 85 to 569 individuals in a recent review of this topic)^28^. Likewise, genetic screening in large cohort studies is often limited to few variants, and those variants are often overrepresented in specific ancestral populations that can confound clinical-genetic associations^11^. Clinical testing, historically a large source of genetic information, is often limited to certain populations (young onset or ancestral populations) limiting the implications beyond these groups. Despite this limitation, prior research and clinical genetic screening efforts have reported differences in age at onset, rate of disease progression, and motor and non-motor complications in *GBA1* and *LRRK2* carriers compared to non-carriers^32,33^. An unbiased approach to genetic and molecular characterization limits ascertainment bias and improves external validity for these prior studies. Thus, the clinic-wide capture utilizing multiple gene variant screening afforded by the MIND study greatly amplifies the importance of these sex- and gene-based differences. Moreover, differences are small, such that it may be difficult to predict based on phenotypic characteristics which individual PD patients carry *LRRK2* or *GBA1* variants. Thus, global screening may be preferred, and our study demonstrates feasibility.

We acknowledge our study’s limitations. First, while we have captured greater than 50% of our entire clinic population, it is still possible that the MIND study suffers from selection bias. We note, however, that frequency for sex (36% women) among MIND enrollees does not differ from frequencies for the total clinic population (38% women for 3010 possible PD patients seen at the PDMDC during MIND enrollment period). Regarding race, while we have more than doubled the representation of participants self-identifying as non-White, compared to historical Penn-based PD cohorts (9% compared to 4%), 21% of PDMDC patients identify as non-White, representing a potential for bias in these results as well as an opportunity for ongoing enrollment to address this disparity. Second, clinical features described in this study are by self-report only, which could impact some of our findings. For example, *GBA1* carriers were not more likely to report cognitive impairment or psychosis in this study, deviating from prior reports^34^. In contrast, our significant findings relating to anxiety, for example, pertain to subjective, but important, symptoms, thus increasing the likelihood that our survey-based approach captures accurate information. Third, as a single-site study, it is unclear whether the high efficiency in enrollment seen here will be widely reproducible. We note, however, that the PDMDC clinical site encompasses the practices of 19 movement disorders specialists, and more than one-third of the MIND cohort enrollment period was impacted by the COVID-19 pandemic, offering confidence that rates of uptake for the global capture approach reported here are robust to practice style and can withstand even an unforeseen, once-in-a-century event.

In summary, we demonstrate that a clinic-wide approach to capturing genetic and clinical data at the time of the clinical visit is feasible in a busy clinical setting. As such, the MIND study offers a model for how genetic information might enter the clinic at scale.

## Methods

### Participant recruitment and enrollment

This study was approved by the University of Pennsylvania Institutional Review Board, and informed consent was obtained at study enrollment. Clinical research coordinators screened the electronic medical record (EMR) and called eligible patients in advance of the PD&MDC visit. Patients were recruited from the movement disorders clinic at the Parkinson’s Disease and Movement Disorders Center (PDMDC) and enrolled on the day of their clinical visit. Patients were approached for enrollment at their office visit if they met the following inclusion criteria: (1) greater than or equal to 21 years of age, (2) a clinical diagnosis of PD by a movement disorder specialist, and (3) able to provide informed consent. Patients were excluded if they were unable to consent to research or were a part of a vulnerable population (e.g., pregnant and lactating women, prisoners). All patients with PD seen at the PDMDC were eligible if they met the inclusion and exclusion criteria above. Participants completed one 20–30-min study visit, where patients reviewed and signed an informed consent form, completed a brief clinical questionnaire, and provided a blood or saliva sample. The informed consent form included three optional consent statements that allowed the study staff to: (1) access the EMR for future research, (2) contact participants to offer additional research studies, and (3) store plasma and DNA as part of the UPenn Integrated Neurodegenerative Disease Database biobank. Due to the SARS-CoV-2 pandemic or by preference, some participants were enrolled remotely beginning September 2020: coordinators called eligible patients around the time of their telehealth visit, and if the patient was interested, they scheduled a 20–30-minute virtual study appointment. A saliva collection kit with pre-paid return shipment materials was sent to the patient’s home address. Patients met with the study staff through a secure audio video platform (Bluejeans or Zoom), reviewed and signed an electronic version of the informed consent form in REDCap, answered the clinical questionnaire, and completed an observed saliva collection. The saliva sample was securely packaged in a return envelope and returned to the study team for DNA extraction and genetic testing.

### Study visit and electronic medical record extraction

Trained clinical research coordinators conducted the study visits as previously described^18^. Participants completed a questionnaire to ascertain demographic information, family history, and clinical symptoms, as previously described^18^. Data were directly entered into a REDCap electronic database hosted at UPenn^18^ or by paper at participant request. For all participants that allowed EMR access, medication records were extracted by matching the medical record number to determine current use of PD-related medications. Blood was drawn by peripheral venipuncture into sterile EDTA vacutainer tubes and processed on the day of collection according to previously published protocols^35^. Briefly, 4 ml blood were used for DNA extraction, and remaining blood was centrifuged, and plasma was aliquoted and frozen at –80 °C for future studies. Due to the SARS-CoV-2 pandemic or by preference, some participants were enrolled remotely by providing a saliva sample beginning in September 2020. Saliva samples were obtained in an Oragene-DNA OG 500 collection kit (DNA Genotek, Ontario Canada) and incubated at 55 °C for at least 2 h up to overnight to inactivate nucleases. DNA was extracted in a semi-automated QuickGene 610 L using the DNA whole blood kit following the manufacturer’s protocol (Autogen, Holliston, MA). For all participants who allowed EMR access, medication records were extracted by matching the medical record number to determine use of PD-related medications at the time of the enrollment visit.

### Genetic testing

All participants were screened for 14 *GBA1* and 8 *LRRK2* variants on an expanded MIND assay (v2, Supplementary Table 1). The assay was designed as described previously with modifications to add 10 additional *GBA1* variants^18^. Briefly, twenty-two SNPtype assays were designed by D3 Assay Design tool (Standard BioTools, San Francisco, CA). Allele-specific PCR was performed using either Flex Six or 48.48 genotyping Dynamic Array integrated fluidic circuits (IFCs) (Standard BioTools), and genotyping was carried out using the Biomark™ HD system (Standard BioTools) according to the manufacturer’s protocol. Specific target amplification (STA) was performed to enrich target sequences flanking the 22 SNPs using 40 ng genomic DNA and 50 nM of each STA and locus-specific primer pairs. Pre-amplified DNA was mixed with 2X Fast Probe Master mix (Biotium Inc., Fremont, CA), 20X SNP Type sample loading reagents, 60X SNP Type reagent, and ROX in 5 μl. Sample mix and 10X SNP Type assay mix was added into assay and sample inlets on the IFC, respectively, and loaded and mixed in the IFC controller. PCR was performed on the BioMark HD System under the following cycling conditions: thermal mix for 30 min at 70 °C followed by 10 min at 25 °C, hot start for 5 min at 95 °C, touchdown PCR for 15 s at 95 °C, 45 s from 64 °C–61 °C dropping 1 °C per cycle, and 15 s at 72 °C, and additional 34 cycles of 15 s at 95 °C, 45 s at 60 °C, and 15 s at 72 °C, and cooling for 10 s at 25 °C. The genotype call data were analyzed using BioMark Genotyping Analysis software which calculated the FAM and VIC relative fluorescence intensities and automatically called genotypes by the k-means clustering algorithm. Automatic genotype calls were reviewed in individual plots after cluster analysis. Positive calls were confirmed by either Taqman SNP genotyping assay or Sanger sequencing.

### Statistical analysis

Statistical analysis was performed in Stata and in R-Studio^36^. Frequencies between groups were compared using χ2 or Fisher Exact Tests, while continuous variables were compared using the non-parametric Kruskal–Wallis Test. Benjamini-Hochberg correction for multiple testing was applied to all analyses. We chose the Benjamini-Hochberg procedure because it limits the false discovery rate, and the Bonferroni method is likely too conservative for outcomes that are not entirely independent^37^.

### Supplementary information

Supplementary Materials

DeidentifiedDataset

## Data Availability

The datasets used and/or analyzed during the current study are found in the Supplementary and have been submitted to DbGaP (https://submit.ncbi.nlm.nih.gov/dbgap/regsys/54461/). The full protocol and participant informed consent form are available on Nature Protocol Exchange^38^. Additional supporting materials including standard operating procedures are available from the corresponding author on reasonable request.

## Code availability

The R code used is found in the Supplementary.
