# Supplementary material for: Genetic and phenotypic characterization of Parkinson’s disease at the clinic-wide level
Source: NPJ Parkinsons Dis. 2024 May 3;10:97. doi: 10.1038/s41531-024-00690-6 (PMC11068880; doi:10.1038/s41531-024-00690-6)
Supplement: Supplementary file 1 — Supplementary Materials [file 41531_2024_690_MOESM1_ESM.pdf]

Supplement for “Genetic and phenotypic characterization of Parkinson’s disease at the clinic-wide level” by Tropea et al. prepared for *NPJ Parkinson’s Disease.*

Supplementary Table 1. *GBA1* and *LRRK2* variants included in testing.

	Target Gene	Coding	Protein	rs ID	GnomAD frequency	MIND frequency
	LRRK2	c.6055G>A	p.Gly2019Ser	rs34637584	0.000488	0.014123
	LRRK2	c.7153G>A	p.Gly2385Arg	rs34778348	0.001680	0.000901
	LRRK2	c.4309A>C	p.Asn1437His	rs74163686	Absent	Absent
	LRRK2	c.4321C>G	p.Arg1441Gly	rs33939927	0.000004	Absent
	LRRK2	c.4321C>T	p.Arg1441Cys	rs33939927	0.000012	Absent
	LRRK2	c.4322G>A	p.Arg1441His	rs34995376	0.000032	Absent
	LRRK2	c.5096A>G	p.Tyr1699Cys	rs35801418	Absent	Absent
	LRRK2	c.6059T>C	p.Ile2020Thr	rs35870237	Absent	Absent
Risk	*GBA1*	c.1093G>A	p.Glu365Lys	rs2230288	0.010730	0.016526
	GBA1	c.1223C>T	p.Thr408Met	rs75548401	0.006124	0.01262
Mild	*GBA1*	c.1226A>G	p.Asn409Ser	rs76763715	0.002235	0.012921
	GBA1	c.1604G>A	p.Arg535His	rs75822236	0.000169	0.000601
	GBA1	c.1304A>C	p.Asn435Thr	rs75385858	Absent	Absent
Severe	*GBA1*	c.1342G>C	p.Asp448His	rs1064651	0.000135	0.001202
	GBA1	c.1448T>C	p.Leu483Pro	rs421016	0.001226	0.003606
	GBA1	c.882T>G	p.His294Gln	rs367968666	0.000219	0.000901
	GBA1	c.115+1G>A		rs104886460	0.000071	0.000601
	GBA1	c.887G>A	p.Arg296Gln	rs78973108	0.000035	0.0003
	GBA1	c.1504C>T	p.Arg502Cys	rs80356771	0.000067	0.0003
	GBA1	c.475C>T	p.Arg159Trp	rs439898	Absent	Absent
	GBA1	c.680A>G	p.Asn227Ser	rs364897	0.000074	Absent
	GBA1	c.1297G>T	p.Val433Leu	rs80356769	0.000032	Absent

1 case identified as RecNcil (comprised of variants: p.L483P, p.A495P, and p.Val499=)

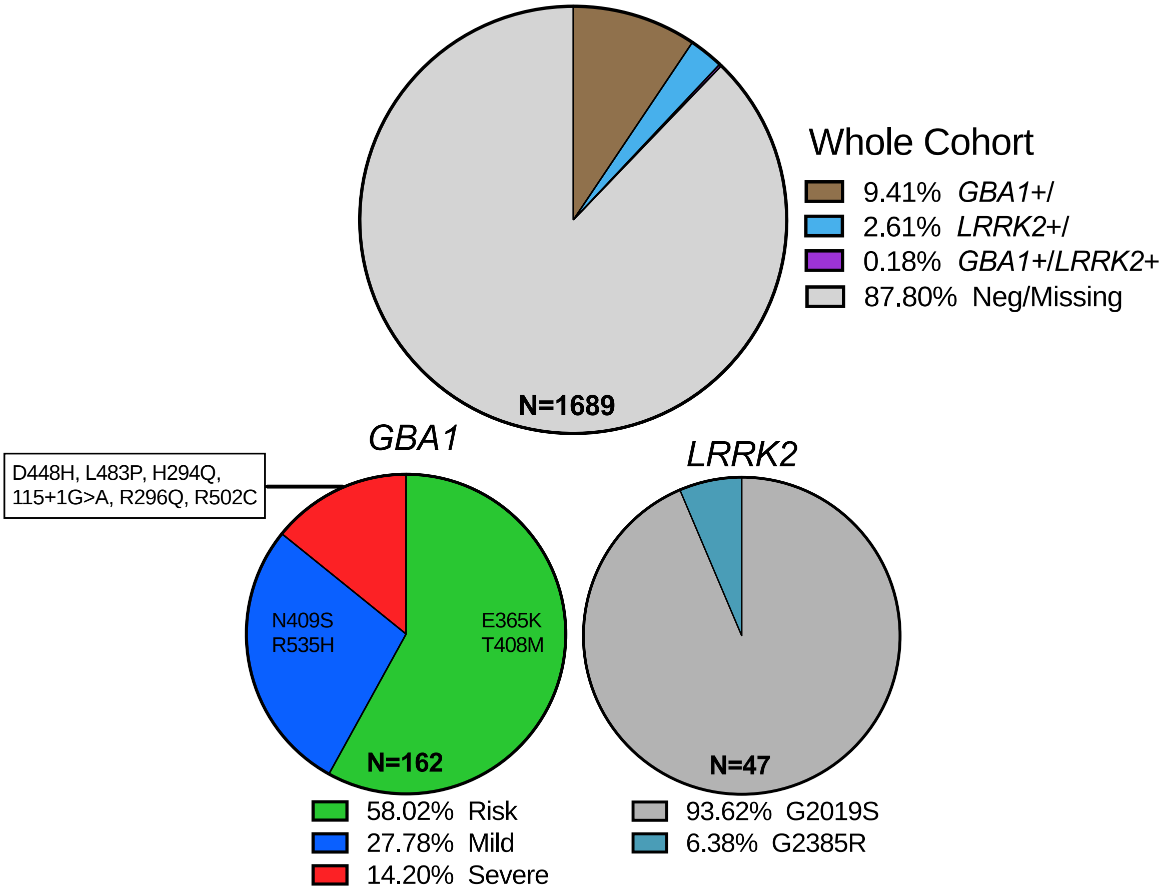

Supplemental Figure 1. GBA1 and LRRK2 testing results in the Molecular Integration in Neurological Diagnosis Initiative Parkinson’s Disease Cohort.

Supplemental Table 2. *GBA1* and *LRRK2* Testing Results.

Target Gene	Variant	N (HET/HOM)
LRRK2	G2019S	41/3
	G2385R	3/0
GBA1	E365K	53/1
	T408M	40/1
	N409S	41/1
	R535H	2/0
	D448H	4/0
	L483P	12/0
	H294Q	3/0
	115+1G>A	2/0
	R296Q	1/0
	R502C	1/0
Complex	*GBA1* Rec1	1/0
	LRRK2 G2019S HET/ *GBA1* N409S HET	2
	LRRK2 G2019S HOM/ *GBA1* N409S HET	1
	GBA1 H294Q HET/ *GBA1* D448H HET	1

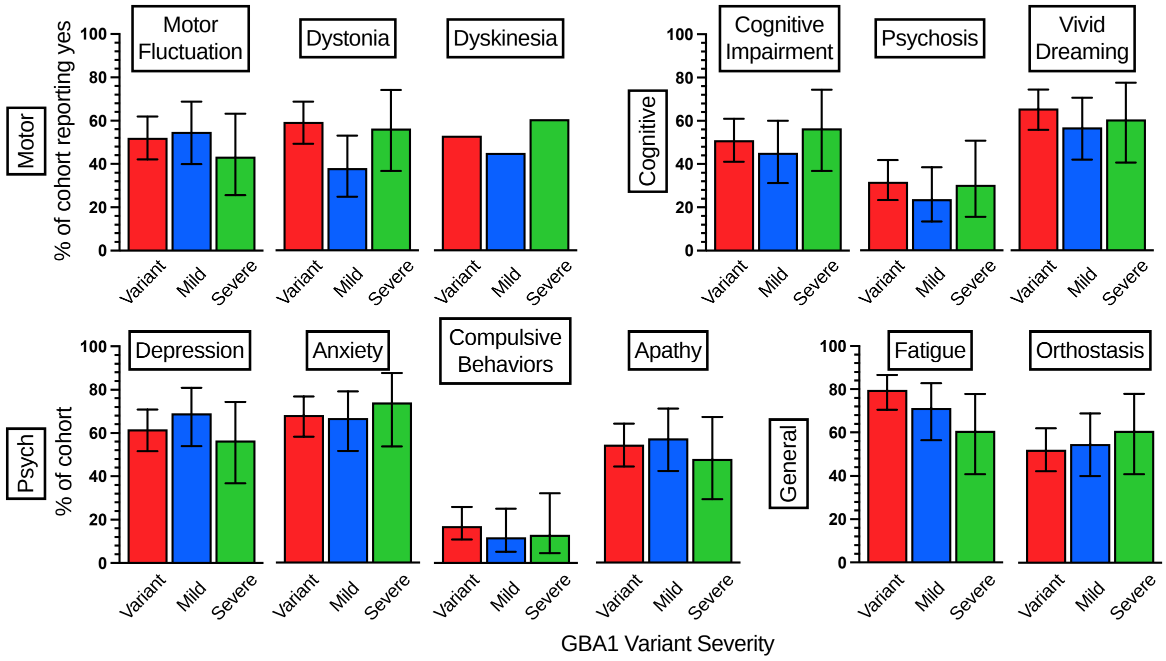

Supplemental Figure 2. Motor and Non-Motor Parkinson's Disease Symptoms by *GBA1* Variant Severity. Responses groups as ever (currently or in the past) or never experiencing a symptom. Heterozygote and homozygote carriers are combined. Error bars represent 95%CI.

R Code

```
#Loading Packages
library(readxl)
library(kableExtra)
library(vtable)
library(epiDisplay)
library(psych)
library(crosstable)
library(gmodels)

###Summary statistics for the whole cohort
###Data for Table 1 and Figure 2 (frequency of motor and non-motor symptoms)
sumtable(MINDFinal)
tab1(MINDFinal$Race)
sumtable(MINDFinal[MINDFinal$GBAYN == 0 & MINDFinal$LRRK2YN == 0, ])
tab1(MINDFinal[MINDFinal$GBAYN == 0 & MINDFinal$LRRK2YN == 0, ]$Race)
sumtable(MINDFinal[MINDFinal$GBAYN == 1 & MINDFinal$LRRK2YN == 0, ])
tab1(MINDFinal[MINDFinal$GBAYN == 1 & MINDFinal$LRRK2YN == 0, ]$Race)
sumtable(MINDFinal[MINDFinal$GBAYN == 0 & MINDFinal$LRRK2YN == 1, ])
tab1(MINDFinal[MINDFinal$GBAYN == 0 & MINDFinal$LRRK2YN == 1, ]$Race)

###gene group comparisons
###GBA
sumtable(MINDGBA)
table(MINDGBA$GBAYN, MINDGBA$Sex)
fisher.test(table(MINDGBA$GBAYN, MINDGBA$Sex))
table(MINDGBA$GBAYN, MINDGBA$Ethnicity)
table(MINDGBA$GBAYN, MINDGBA$Race)
describeBy(MINDGBA$AgeEnrollment, group=MINDGBA$GBAYN)
table(MINDGBA$GBAYN, MINDGBA$Onset)
kruskal.test(MINDGBA$AgeDiag, MINDGBA$GBAYN)
kruskal.test(MINDGBA$AgeEnrollment, MINDGBA$GBAYN)
fisher.test(table(MINDGBA$GBAYN, MINDGBA$Onset))
fisher.test(table(MINDGBA$GBAYN, MINDGBA$FHSum))

#LRRK2
sumtable(MINDLRRK2)
table(MINDLRRK2$LRRK2YN, MINDLRRK2$Sex)
fisher.test(table(MINDLRRK2$LRRK2YN, MINDLRRK2$Sex))
table(MINDLRRK2$LRRK2YN, MINDLRRK2$Ethnicity)
table(MINDLRRK2$LRRK2YN, MINDLRRK2$Race)
describeBy(MINDLRRK2$AgeEnrollment, group=MINDLRRK2$LRRK2YN)
table(MINDLRRK2$LRRK2YN, MINDLRRK2$Onset)
table(MINDLRRK2$LRRK2YN, MINDLRRK2$FHSum)
kruskal.test(MINDLRRK2$AgeDiag, MINDLRRK2$LRRK2YN)
kruskal.test(MINDLRRK2$AgeEnrollment, MINDLRRK2$LRRK2YN)
fisher.test(table(MINDLRRK2$LRRK2YN, MINDLRRK2$Onset))
fisher.test(table(MINDLRRK2$LRRK2YN, MINDLRRK2$FHSum))

##p-value correction Table 1
#Sex
```

```
Sexgenepvalues = c(0.08123, 0.02407)
p.adjust(Sexgenepvalues, method = "BH")

#Age Diagnosis
GBADEMAGEpvalues = c(0.1077,0.3473)
p.adjust(GBADEMAGEpvalues, method = "BH")

#Age Enrollment
GBADEMenrollpvalues = c(0.02493,0.6828)
p.adjust(GBADEMenrollpvalues, method = "BH")

#Clinical Presentation
GBADEMpresentpvalues = c(0.4821,0.4158)
p.adjust(GBADEMpresentpvalues, method = "BH")

#1st degree fam
GBADEMFHpvalues = c(0.2258,0.00107)
p.adjust(GBADEMFHpvalues, method = "BH")


###summary of clinical features
###yes or no with comparisons
###Sex
crosstable(MINDFinal, c(Dyskinesia1, Dystonia1, MotorFluc1, CognitiveImpair1,
CompulsiveBehav1, Depression1, Lightheaded1, Anxiety1, Fatigue1, Psychosis1,
DecreasedMot1, VivdDreaming1), test = TRUE, effect = TRUE, by = Sex01) %>%
as_flextable(keep_id=TRUE)

###Sex-corrected pvalues
Sexpvalues = c(0.3232, 0.0034, 0.9887, 0.0003, 0.2111, 0.0254, 0.0341, 0.0002, 0.4032,
0.9756, 0.5116, 0.0040)
print("Sex")
p.adjust(Sexpvalues, method = "BH")

###GBA
crosstable(MINDGBA, c(Dyskinesia1, Dystonia1, MotorFluc1, CognitiveImpair1,
CompulsiveBehav1, Depression1, Lightheaded1, Anxiety1, Fatigue1, Psychosis1,
DecreasedMot1, VivdDreaming1), test = TRUE, effect = TRUE, by = GBAYN) %>%
as_flextable(keep_id=TRUE)

###GBA-corrected pvalues
GBApvalues = c(0.0529, 0.1556, 0.7982, 0.2553, 0.9666, 0.000033, 0.3559, 0.0060, 0.1004,
0.0135, 0.0537, 0.1708)
p.adjust(GBApvalues, method = "BH")


#GBASev
crosstable(MINDGBASev, c(Dyskinesia1, Dystonia1, MotorFluc1, CognitiveImpair1,
CompulsiveBehav1, Depression1, Lightheaded1, Anxiety1, Fatigue1, Psychosis1,
DecreasedMot1, VivdDreaming1), test = TRUE, effect = TRUE, by = GBASev) %>%
as_flextable(keep_id=TRUE)
```

```
###GBA-corrected pvalues
GBApvalues = c(0.0529, 0.1556, 0.7982, 0.2553, 0.9666, 0.000033, 0.3559, 0.0060, 0.1004,
0.0135, 0.0537, 0.1708)
p.adjust(GBApvalues, method = "BH")

###LRRK2
crosstable(MINDLRRK2, c(Dyskinesia1, Dystonia1, MotorFluc1, CognitiveImpair1,
CompulsiveBehav1, Depression1, Lightheaded1, Anxiety1, Fatigue1, Psychosis1,
DecreasedMot1, VivdDreaming1), test = TRUE, effect = TRUE, by = LRRK2YN) %>%
as_flextable(keep_id=TRUE)

###LRRK2-corrected pvalues
LRRK2pvalues = c(0.2862, 0.1306, 0.8173, 0.0706, 0.0256, 0.3801, 0.0697, 0.5110, 0.4933,
0.4095, 0.4150, 0.0141)
print("LRRK2")
p.adjust(LRRK2pvalues, method = "BH")

###adjusted pvalues
GBApvalues = c(0.054, 0.157, 0.803, 0.277, 1, 0, 0.36, 0.007, 0.105, 0.015, 0.055, 0.17)
print("GBA")
p.adjust(GBApvalues, method = "BH")

LRRK2pvalues = c(0.356, 0.168, 0.879, 0.09, 0.033, 0.443, 0.091, 0.539, 0.297, 0.572, 0.445,
0.02)
print("LRRK2")
p.adjust(LRRK2pvalues, method = "BH")

Sexpvalues = c(0.333, 0.004, 1, 0, 0.231, 0.026, 0.033, 0, 0.411, 0.951, 0.542,0.004)
print("Sex")
p.adjust(Sexpvalues, method = "BH")

MedSexpvalues = c(0.006, 0.078, 0.562, 0.262, 0.305, 0.346, 0.00000556, 0.359, 0.291, 0.041)
print("Meds.Sex")
p.adjust(MedSexpvalues, method = "BH")

MedGBApvalues = c(0.087, 0.189, 0.701, 0.217, 0.525, 1, 0.066, 0.259, 0.354, 0.385)
print("Meds.GBA")
p.adjust(MedGBApvalues, method = "BH")

MedLRRK2pvalues = c(0.513, 0.234, 0.279, 1, 0.343, 0.256, 0.175, 1, 0.009, 0.819)
print("Meds.LRRK2")
p.adjust(MedLRRK2pvalues, method = "BH")
```
